# Mevalonate Cascade Inhibition by Simvastatin Induces the Intrinsic Apoptosis Pathway via Depletion of Isoprenoids in Tumor Cells

**DOI:** 10.1038/srep44841

**Published:** 2017-03-27

**Authors:** Javad Alizadeh, Amir A. Zeki, Nima Mirzaei, Sandipan Tewary, Adel Rezaei Moghadam, Aleksandra Glogowska, Pandian Nagakannan, Eftekhar Eftekharpour, Emilia Wiechec, Joseph W. Gordon, Fred. Y. Xu, Jared T. Field, Ken Y. Yoneda, Nicholas J. Kenyon, Mohammad Hashemi, Grant M. Hatch, Sabine Hombach-Klonisch, Thomas Klonisch, Saeid Ghavami

**Affiliations:** 1 Department of Human Anatomy and Cell Science, Max Rady College of Medicine, Rady Faculty of Health Sciences, University of Manitoba, Winnipeg, Canada; 2 Biology of Breathing Theme, Children’s Hospital Research Institute of Manitoba, University of Manitoba, Winnipeg, Canada; 3 Division of Pulmonary, Critical Care, and Sleep Medicine, Department of Internal Medicine, Center for Comparative Respiratory Biology and Medicine, Davis, CA, USA; 4 Department of Physiology and Pathophysiology, Regenerative Medicine, Program and Spinal Cord research Center, Max Rady College of Medicine, Rady Faculty of Health Sciences, University of Manitoba, Winnipeg, Canada; 5 Department of Clinical and Experimental Moledicine, Division of Otorhinolaryngology, Linköping University, 581-85, Linköping, Sweden; 6 College of Nursing and Children’s Hospital Research Institute of Manitoba, Rady Faculty of Health Sciences, University of Manitoba, Winnipeg, Canada; 7 DREAM, Children’s Hospital Research Institute of Manitoba, Center for Research and Treatment of Atherosclerosis and Department of Pharmacology and Therapeutics, University of Manitoba, Winnipeg, Canada; 8 Department of Biological Sciences, Faculty of Science, University of Manitoba, Winnipeg, Canada; 9 Department of Clinical Biochemistry, Zehedan University of Medical Sciences, Zahedan, Iran; 10 Health Policy Research Centre, Shiraz University of Medical Sciences, Shiraz, Iran

## Abstract

The mevalonate (MEV) cascade is responsible for cholesterol biosynthesis and the formation of the intermediate metabolites geranylgeranylpyrophosphate (GGPP) and farnesylpyrophosphate (FPP) used in the prenylation of proteins. Here we show that the MEV cascade inhibitor simvastatin induced significant cell death in a wide range of human tumor cell lines, including glioblastoma, astrocytoma, neuroblastoma, lung adenocarcinoma, and breast cancer. Simvastatin induced apoptotic cell death via the intrinsic apoptotic pathway. In all cancer cell types tested, simvastatin-induced cell death was not rescued by cholesterol, but was dependent on GGPP- and FPP-depletion. We confirmed that simvastatin caused the translocation of the small Rho GTPases RhoA, Cdc42, and Rac1/2/3 from cell membranes to the cytosol in U251 (glioblastoma), A549 (lung adenocarcinoma) and MDA-MB-231(breast cancer). Simvastatin-induced Rho-GTP loading significantly increased in U251 cells which were reversed with MEV, FPP, GGPP. In contrast, simvastatin did not change Rho-GTP loading in A549 and MDA-MB-231. Inhibition of geranylgeranyltransferase I by GGTi-298, but not farnesyltransferase by FTi-277, induced significant cell death in U251, A549, and MDA-MB-231. These results indicate that MEV cascade inhibition by simvastatin induced the intrinsic apoptosis pathway via inhibition of Rho family prenylation and depletion of GGPP, in a variety of different human cancer cell lines.

Tumor cells undergo significant metabolic reprogramming to serve their increased needs for energy and macromolecules^1^,^2^. Cholesterol is essential for eukaryotic cells and critical for the propagation of tumor cells which utilize *de novo* cholesterol biosynthesis via fatty acid synthesis and the mevalonate (MEV) cascade^3^,^4^,^5^. The MEV pathway also generates non-sterol end products called isoprenoids, including isopentenyl pyrophosphate, farnesyl and geranylgeranyl isoprenoids, dolichol, ubiquinone, and isopentenyl adenine^5^,^6^. In eukaryotes, the MEV pathway is the only metabolic pathway capable of generating the isoprenoids FPP and GGPP. In tumor cells, the MEV metabolism is dysregulated^7^,^8^ and glucose, glutamine, and acetate serve as substrates to fuel an anabolic MEV pathway^5^,^9^,^10^. 3-hydroxy-3-methylglutaryl-CoA reductase (HMGCR) is the rate-limiting enzyme in the biosynthesis of MVA and cholesterol. Considered a new member of the family of metabolic oncogenes^11^, this key enzyme is frequently dysregulated in tumor cells^12^,^13^,^14^,^15^.

Statin drugs inhibit HMGCR and this can modulate several cellular signaling pathways relevant to tumor formation, including angiogenesis, cellular proliferation, cell cycle regulation, gene expression, metastatic potential, and cell death^5^,^16^,^17^,^18^. Statins deplete cellular FPP, GGPP, and cholesterol by decreasing MEV levels^5^,^19^,^20^, cause cell cycle arrest by up-regulation of cyclin-dependent kinase inhibitors p21^21^ and p27^22^,^23^, induce apoptosis^21^,^24^,^25^,^26^ and modulate proteasome activity^27^,^28^. While statin-mediated inhibition of small Rho GTPase prenylation (geranylgeranylation and farnesylation) and cholesterol depletion are important contributing factors, the exact mechanisms of statin-induced apoptosis in tumor cells has not yet been fully elucidated.

The increased demand on the MEV pathway and its products during enhanced proliferation sensitizes tumor cells to inhibition of the MEV pathway by statins. Several recent epidemiological investigations confirmed a possible role of statins as anti-cancer agents^29^,^30^,^31^. In recent phase II clinical trials, lovastatin or pravastatin only showed limited inhibition of tumor growth^21^,^32^. However, their combination with different chemotherapeutic drugs produced more effective anti-tumor effects in several preclinical models^32^,^33^. According to www.clinicaltrials.gov there are approximately 153 clinical trials evaluating the use of statins as adjunctive or co-treatment for various types of cancers. The most common statins used in these studies is simvastatin (53) followed by atorvastatin (37), rosuvastatin (21), pravastatin (18), lovastatin (11), and fluvastatin (3). This adds up to 143 studies, and the remaining 10 studies are looking at “statin use” and are inclusive of all statins.

This raises the important question of which cancers, which statins, and with which chemotherapeutic or radiation combination will we produce the desired result of selective or preferential cancer cell apoptosis while reducing mortality. Therefore, elucidating these mechanisms remains of paramount importance if we hope to develop potentially more specific and effective targeted therapies based on the anti-cancer effects of statins. Our present study provides promising avenues to pursue this line of thinking.

In the present study, we investigated the ability of simvastatin to induce apoptosis in a broad range of human tumor cell lines of different origin. We hypothesized that statin-induced depletion of prenylation intermediates in the mevalonate cascade is responsible for apoptosis in cancer cells. Here we show that simvastatin-induced apoptosis was independent of cholesterol but required the prenylation intermediates GGPP and FPP.

## Results

### Simvastatin induces concentration- and time-dependent cell death in human brain, lung, and breast cancer cell lines

**Figure 1:**
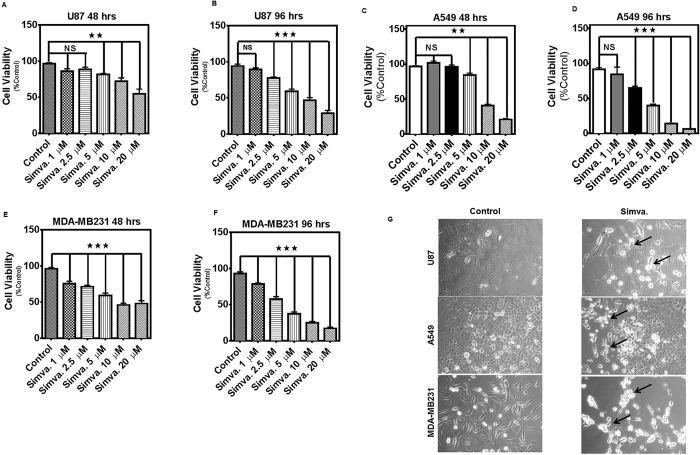
Simvastatin induces cell death in glioblastoma, non-small lung cancer cell, and breast cancer cell lines. (**A**,**B**) U87 cells were treated with simvastatin (1, 2.5, 5, 10 or 20 μM) and cell viability was assessed 48 and 96 hrs thereafter by MTT assay. Control cells for each time point were treated with the solvent control (DMSO). Results are expressed as percentage of corresponding time point control and represent the means ± SD of 15 replicates in three independent experiments (**P < 0.01; ***P < 0.001). (**C**,**D**) A549 cells were treated with simvastatin (1, 2.5, 5, 10 or 20 μM) and cell viability was assessed 48 and 96 hrs thereafter by MTT assay. Control cells for each time point were treated with the solvent control (DMSO). Results are expressed as percentage of corresponding time point control and represent the means ± SD of 15 replicates in 3 independent experiments (**P < 0.01; ***P < 0.001). (**E**,**F**) MDA-MB-231 cells were treated with simvastatin (1, 2.5, 5, 10 or 20 μM) and cell viability was assessed 48 and 96 hrs thereafter by MTT assay. Control cells for each time point were treated with the solvent control (DMSO). Results are expressed as percentage of corresponding time point control and represent the means ± SD of 15 replicates in three independent experiments (***P < 0.001). (**G**) U87, A549, and MDA-MB231 cells treated with 10 μM simvastatin for 60 hrs were then photographed under phase contrast microscopy settings. Arrows indicate partially detached cells with condensed morphology.

We initially tested the concentration (0–20 μM) and time (0–96 h) effects of simvastatin on viability of glioblastoma (U87 (ATCC- HTB-14™), U251 (ATCC)), neuroblastoma (SH-SY5Y (ATCC-CRL-2266™)), lung adenocarcinoma (A549 (ATCC- CCL-185™), H460 (ATCC- HTB-177™), H1650 (ATCC-CRL-5883™), H1975 (ATCC-CRL-5908™)), breast cancer (MCF-7 (ATCC-HTB-22™), MDA-MB-231 (ATCC-HTB-26™)), human astrocyte (Sciencell-1800), human HBE1(Gift from Dr. Amir Zeki lab, UC Davis), and human MCFD10A (Gift from Dr. Amir Zeki lab, UC Davis) (Fig. 1A–F) (Supplementary Fig. 1A–S). Simvastatin induces significant cell death in U87 (Fig. 1A,B) [(48 h, *P* < *0.01* for concentrations ≥5 μM), (96 h, *P* < *0.001* for concentrations ≥2.5 μM)], U251 (Fig. 1C,D) [(48 h, 96 h *P* < *0.001* for concentrations ≥2.5 μM)], SH-SY5Y (Fig. 1E,F) [(96 h, *P* < *0.001* for concentrations ≥2.5 μM)], A549 (G, H) [(48 h, *P* < *0.01* for concentrations ≥5 μM), (96 h, *P* < *0.001* for concentrations ≥2.5 μM)], H460 (I, J) [(48 h, *P* < *0.01* for concentrations ≥10 μM), (96 h, *P* < *0.001* for concentrations ≥2.5 μM)], H1650 (K, L) [(48 h, *P* < *0.01* for concentrations ≥5 μM), (96 h, *P* < *0.001* for concentrations ≥1 μM)], H1975 (M, N) [(48 h, *P* < *0.01* for concentrations ≥5 μM), (96 h, *P* < *0.001* for concentrations ≥1 μM)], MCF-7 (O, P) [(48 h, *P* < *0.01* for concentrations ≥2.5 μM), (96 h, *P* < *0.001* for concentrations ≥2.5 μM)], MDA-MB-231 (Q, R) [(48 h, *P* < *0.001* for concentrations ≥1 μM), (96 h, *P* < *0.0001* for concentrations ≥1 μM)]. The morphological changes of U87, A549, and MDA-MB213 cells observed when treated with simvastatin (10 μM, 60 h) are shown in Fig. 1G. Our results also showed that simvastatin induced both time- and concentration-dependent significant cell death (P < *0.0001*) in human astrocytes (Supplementary Figure 1E and F), HBE1 (human bronchial epithelial cells) (Supplementary Figure 1M and N), and MCF10A (non-tumorigenic epithelial breast cell line) (Supplementary Figure 1Q and S). In summary, simvastatin induced a concentration- and time-dependent cell death in a broad range of human tumor and non-malignant cell lines.

### Simvastatin induces the intrinsic apoptotic pathway in human cancer cells

**Figure 2:**
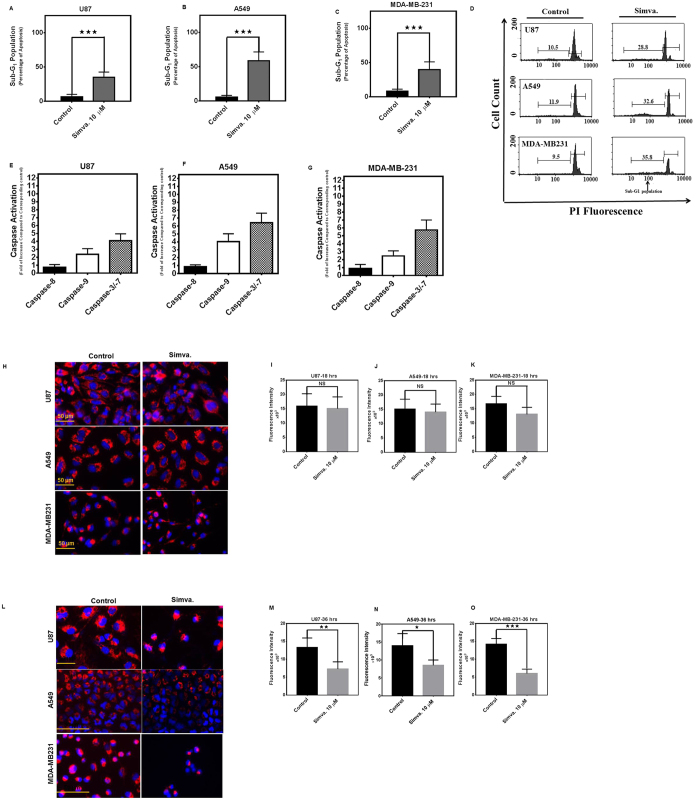
Simvastatin induces intrinsic apoptosis in glioblastoma, non-small lung cancer cell, and breast cancer cell lines. Percent sub-G1 (**A**) U87, (**B**) A549, (**C**) MDA-MB-231 abundance induced by simvastatin (10 μM) or DMSO solvent control after 60 hrs. Results represent the means ± SD of 9 replicates in three independent experiments. *P < 0.05; and ***P < 0.001 compared to time-matched control. Representative figures of the flow cytometry histogram for U87, A549 and MDA-MB-231 are shown (**D**). Effects of simvastatin (10 μM) treatment (36 hrs) on caspase-8, caspase-3/-7, and caspase-9 enzymatic activity, as detected by Caspase-Glo^®^ luminometric assay in U87 (**E**), A549 (**F**), MDA-MB-231 (**G**). Caspase activity normalized to that measured for solvent-only treated cultures is represented on the Y-axis. The data represent mean ± SD of triplicate experiments performed on 3 independent experiments. U87, A549, and MDA-MB231 cells were treated with 10 μM simvastatin for 18 and 36 hrs. Control cells were treated with media and vehicle control (DMSO). Cells were stained with TMRM, Hoechst, and imaged by standard fluorescence techniques. Simvastatin (10 μM, 18 hrs) did not significantly change TMRM fluorescence intensity in U87, A549, and MDA-MB231 cells (**H**–**K**),while simvastatin (10 μM, 36 hrs) significantly decreased TMRM florescence intensity in U87 (P < 0.01), A549 (P < 0.05), and MDA-MB231 (P < 0.001) (**L**–**O**) which indicates the decrease of mitochondrial membrane potential in simvastatin-treated cells. Data represent the average values from triplicates of three independent experiments.

We confirmed that simvastatin (10 μM, 60 h) significantly induced apoptotic cell death in U87, U251, SH-SY5-Y, A549, H460, H1650, H1975, MCF-7, and MDA-MB-231 cell lines (Fig. 2A–D) (Supplementary Fig. 2A–F). As shown in Fig. 2D, an apoptotic cell population was identified as sub-G_1_ population in flow cytometry as had been described by us previously^27^. Simvastatin (10 μM, 48 h) failed to activate caspase-8 but did activate caspase-9 and caspase-3/-7 in all cell lines tested at 36 h (Fig. 2E–G) (Supplementary Fig. 2G–L). These findings indicated that MEV cascade inhibition induced the intrinsic apoptotic pathways in all tumor cell lines tested. The involvement of the intrinsic apoptosis pathway was further confirmed by measuring mitochondrial membrane potential in simvastatin-treated (10 μM, 36 h) cells (U87, A549, MDA-MB231) (Fig. 2L–O). Simvastatin significantly reduced mitochondrial membrane potential in these tumor cells (U87, *P* < *0.01*, A549, *P* < *0.05*, and MDA-MB231, *P* < *0.001*). We also showed that shorter treatment of simvastatin (10 mΜ, 18 h) did not change mitochondrial membrane potential in these cells (Fig. 2H–K) (*P* > *0.05*).

### Simvastatin-induced cell death in human cancer cells is not rescued by cholesterol but is dependent on depletion of FPP and GGPP

**Figure 3:**
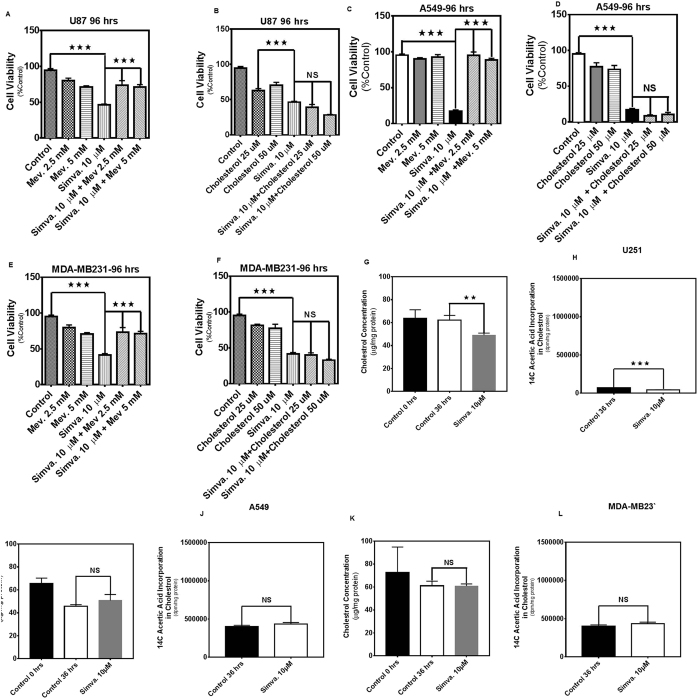
Simvastatin-induced cell death in glioblastoma, non-small lung cancer cell, and breast cancer cell lines is independent of cholesterol. 2.5, and 5 mM MEV or, 25, 50 μM cholesterol, were added to the cells 4 hrs prior to treatment with simvastatin (10 μM, 96 hrs). Cell death was measured by MTT assay in U87 (**A**,**B**), A549 (**C**,**D**), and MDA-MB-231 (**E**,**F**). For each experiment control cells were treated with simvastatin solvent (DMSO) alone (control) or with both DMSO and the appropriate solvent (i.e. ethanol for “mevalonate control”). Results are expressed as mean ± SD of 9 replicate in 3 independent experiments (*P < 0.05, **P < 0.01, and ***P < 0.001). U87, A549, and MDA-MB231 cells were treated with simvastatin (10 μM) and after 36 hrs total and *de novo* cholesterol content in cells were measured. Both total and de novo cholesterol were significantly decreased in U251 cells (**G**,**H**) after simvastatin treatment. However, there was no significant change in the amount of total and *de novo* cholesterol for A549 (**I**,**J**) and MDA-MB231 cells (**K**,**L**). For each experiment control cells were treated with DMSO. Results are expressed as mean ± SD of 3 replicate 3 independent experiments (*P < 0.05, **P < 0.01, and ***P < 0.001).

**Figure 4:**
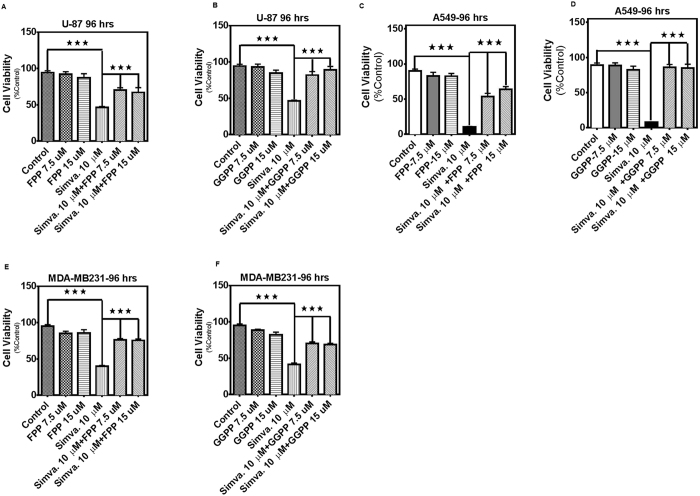
Simvastatin-induced cell death in glioblastoma, non-small lung cancer cell, and breast cancer cell lines dependents on FPP and GGPP. 7.5, 15 μM FPP, or 7.5, 15 μM GGPP were added to the cells 4 hrs prior to treatment with simvastatin (10 μM, 96 hrs) on cell death, measured by MTT assay in U87 (**A**,**B**), A549 (**C**,**D**), and MDA-MB-231 (**E**,**F**). For each experiment control cells were treated with simvastatin solvent (DMSO) alone (control) or with both DMSO and the appropriate solvent for each cholesterol precursor (i.e. DMSO for “FPP control” and “GGPP control”). Results are expressed as mean ± SD of 9 replicate in 3 independent experiments (*P < 0.05, **P < 0.01, and ***P < 0.001).

Statin-induced apoptosis is due to a loss of cell membrane cholesterol and/or depletion of the polyisoprene cholesterol precursors, FPP and GGPP, which are essential lipid anchors for active small GTPase proteins in cells^5^,^34^,^35^. We used different approaches to identify the mechanism by which MEV cascade inhibition caused apoptosis. First, we performed treatment of tumor cells (U87, U251, A549, H460, H1650, H1975, MCF-7, MDA-MB-231) with simvastatin (10 μM, 96 h) in the presence of MEV (2.5, 5 mM), cholesterol (25, 50 μM), and next used FPP (7.5, 15 μM), and GGPP (7.5, 15 μM) pre-treatment and co-treatment in the cell models. MEV (Fig. 3A,C,E and Supplementary Fig. 3A,C,E,G,I,K), FPP (Fig. 4A,C,E, and Supplementary Fig. 4A,C,E,G,I,K), and GGPP (Fig. 4B,D,F, and Supplementary Fig. 4B,D,F,H,J,L) were each able to significantly inhibit (*P* < *0.001*) simvastatin-induced cell death in all cell lines tested. However, cholesterol did not have any significant effect on simvastatin-induced cell death (Fig. 3B,D,F, and Supplementary Fig. 3B,D,F,H,J). Our results also showed that simvastatin significantly decreased total cholesterol (*P* < *0.01*) and *de novo* cholesterol biosynthesis (*P* < *0.001*) in U251 cells (Fig. 3G,H). Simvastatin did not significantly change total cholesterol or de novo cholesterol biosynthesis (*P* > *0.05*) in A549 (Fig. 3I,J) and MDA-MB231 cells (Fig. 3K,L).

### Simvastatin blocks membrane translocation of Rho family small GTPases

**Figure 5:**
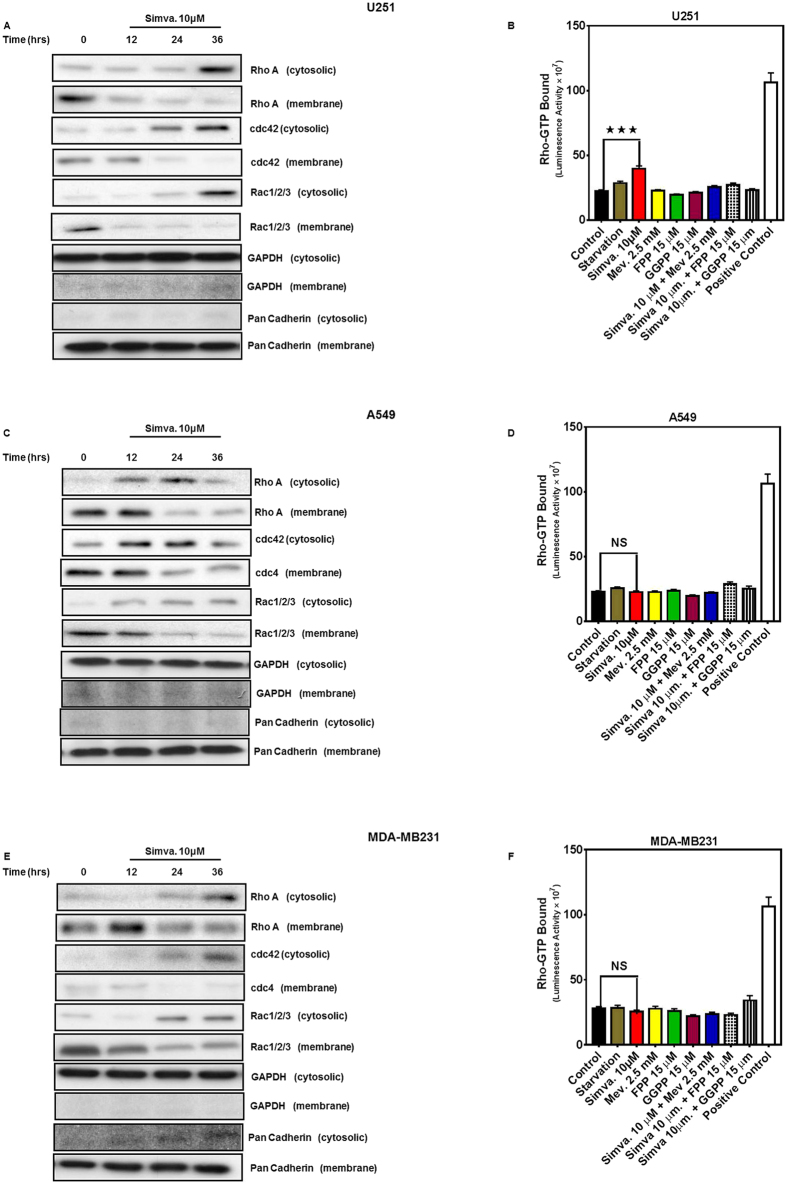
Simvastatin promotes cytosolic localization of RhoA, CDC42, and Rac1/2/3. U87, A549, and MDA-MB231 were treated with simvastatin (10 μM, 12, 24, 36 hrs) and the abundance of RhoA, cdc42, and Rac1/2/3 in membrane and cytosolic fractions obtained from U251 (**A**), A549 (**C**), and MDA-MB-231 (**E**). GAPDH and Pan-Cadherin abundance was also assessed to control for loading in cytosolic and membrane fractions, and to confirm lack of cytosolic contamination in membrane fractions. Data are typical of 3 independent experiments using different primary cultures. Cropped representative of blots have been showed. G-LISA assay was done to evaluate the GTP-bound Rho protein in U251, A549, and MDA-MB231. Different conditions were tested for 36 hrs including starvation, Simva. (10 μM), Mev (2.5 mM), FPP, and GGPP (15 μM), Simva. + Mev, Simva. + FPP, and Simva. + GGPP. Simva. significantly increased GTP-bound Rho in U251 cells (**B**) while Mev, FPP, and GGPP co-treatment decreased GTP-bound Rho compared to control. none of the treatments significantly change GTP-bound protein in A549 (**D**) and MDA-MB231 (**F**) cells. For each experiment a positive control provided in the kit was used and control cells were treated with the reagent in the kit. Results are expressed as mean ± SD of 2 replicates in an independent experiment (***P < 0.001).

We investigated the ability of simvastatin to affect the translocation of small Rho GTPases from the plasma membrane to the cytosol in U251 (Fig. 5A), A549 (Fig. 5C), and MDA-MB231 (Fig. 5E). Simvastatin decreased membrane localization of RhoA, Cdc42, and Rac/1/2/3 and their cytosolic cellular fraction suggesting simvastatin-mediated block in prenylation of small GTPases and poor membrane translocation. We measured GTP-bound Rho protein and showed that simvastatin significantly (*P* < *0.05*) increased GTP-bound Rho in U251 (Fig. 5B), whereas mevalonate, FPP, and GGPP co-treatment decreased GTP-bound Rho to untreated levels. Simvastatin did not significantly change GTP-bound Rho protein in A549 (Fig. 5D) and MDA-MB-231 (Fig. 5F). Thus, simvastatin-induced small GTPase protein prenylation and intracellular localization may vary among different tumor cell lines.

### Inhibitors of Geranygeranyltransferase and Farnesyltransferase Induce Differential Cell Death in Cancer Cells

**Figure 6:**
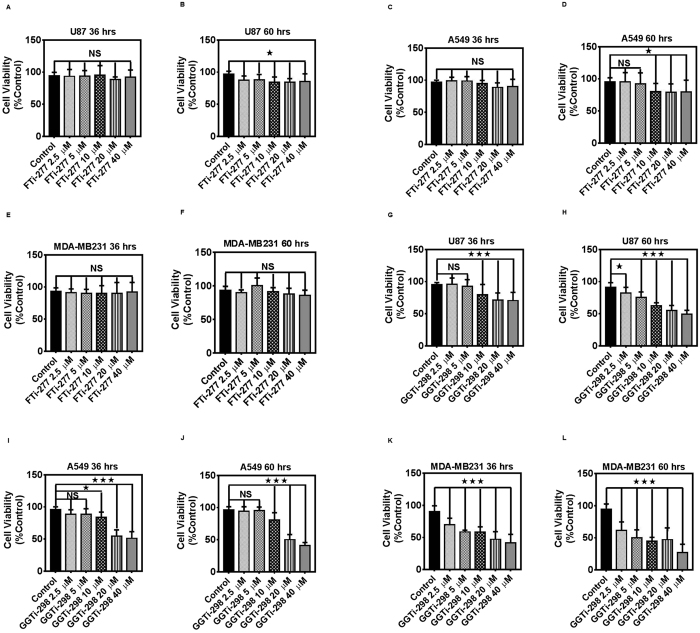
FTi-277 and GGTi-298 induces differential cell death in cancer cells. U87, A549, and MDA-MB231 cells were treated with FTi-277 (0–40 μM, 36, 60 hrs) (**A**–**F**) and GGTi-298 (0–40 μM, 36, 60 hrs) (**G**–**L**) and the cytotoxic effects were measured using MTT assay. For each experiment control cells were treated with GGTi solvent (DMSO) and FTi-277 solvent (distilled water) alone (control). Results are expressed as mean ± SD of 15 replicate in 3 independent experiments (*P < 0.05, **P < 0.01, and ***P < 0.001).

Simvastatin-induced cell death in cancer cells was further investigated using farnesyl transferase inhibitor (FTi-277) and geranylgeranyl transferase I inhibitor (GGTi-298) in U87, A549, and MDA-MB231 cells. FTi-277 (0–40 μM, 36, 60 h) had no significant effect (*P* > *0.05*) on cell death in all three cells in 36 h (Fig. 6A,C,E) and induced less than 15% death in U87 and A549 cells at 60 h (Fig. 6B,D). GGTi-298 (>5 μM) induced significant (*P* > *0.001*) cell death in U87, A549, and MDA-MB231 cells (36 and 60 h, Fig. 6G–L), indicating that gernylgernalytion was the leading mechanism in simvastatin-induced cell death in these cancer cells.

## Discussion

**Figure 7:**
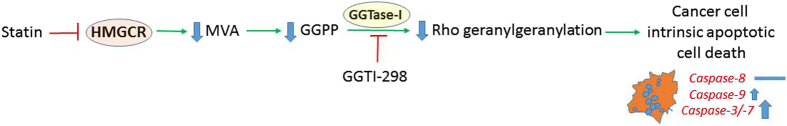
Summary of the mechanism involved in statin-induced cell death in cancer cells. MEV cascade inhibitors induce the intrinsic apoptotic pathway which is regulated by gernaylgenralyation of small Rho GTPAse protein.

In the present study, we found that MEV cascade inhibition by the HMGCR inhibitor simvastatin induced intrinsic apoptosis cell death and decreased mitochondrial membrane potential in a broad range of human tumor cell lines, including glioma (U87, U251), neuroblastoma (SH-SY5Y), lung (A549, H460, H1650, H1975), and breast cancer cells (MCF7, MDA-MB231). Inhibition of the MEV cascade prevented the membrane translocation of the small Rho GTPases RhoA, Cdc42 and Rac1/2/3 and this simvastatin effect was independent of cellular cholesterol but partially rescued by FPP and GGPP. Inhibition of FTase had no effect on cell death and FPP supplementation caused partial reversal of the simvastatin-mediated effects. FPP can also be converted into GGPP and the addition of FPP can restore farnesylation and geranylgeranylation. We also showed that, at least in U251 brain tumor cell line, the membrane localization of prenylated Rho GTPases was important for GTPase activity. Our findings are summarized in Fig. 7.

Simvastatin (10 μM) is known to induce mitochondrial-dependent apoptotic cell death in brain tumor cell lines (U87 and U251) with subsequent activation of caspase-3^36^. Lovastatin (5 μM) can induce GGPP- and FPP-dependent apoptosis in U87 and U251 cells which is regulated by the expression of Bcl2 pro-apoptotic protein (Bim) in the absence of any changes in Blc2 anti-apoptotic protein expression^37^. Interestingly, lovastatin (10 μM)-induced caspase-dependent apoptosis in SH-SY5Y cells was inhibited using caspase-3 (Z-DEVD-FMK) (50 μM), and caspase-9 (Z-LEHD-FMK) (50 μM) inhibitors^37^. Thus, statin mediates its cytotoxic effects via caspases of the intrinsic apoptotic pathway.

Simvastatin also induces pro-apoptotic Bcl2 family members Bcl2 and Bax in MCF-7 and MDA-MB231 cells in a time- and dose-dependent manner^38^,^39^. These effects were also caspase-dependent since simvastatin increased caspase-3 and -9 activity in MDA-MB231 cells^39^. Similarly, fluvastatin and atorvastatin, were also reported to induce dose- and time-dependent apoptosis in MCF-7 and MDA-MB-231 breast cancer cell lines^40^,^41^. Several mechanisms have been proposed for statin-induced cell death in breast cancer cells including an increase of reactive oxygen species (ROS)^42^, the downregulation of survivin expression^43^, increased nitric oxide synthase activity (iNOS or NOS II) via geranylgeranylation^44^, and G1/S cell cycle arrest due to an increase in p21(Waf1/Cip1)^45^.

Simvastatin was shown to induce caspase-dependent apoptosis in A549 cells which is regulated by the Bcl2 family proteins and ROS and can be reversed by treatment with N-acetylcysteine^46^,^47^. Other investigators have reported that simvastatin can induce apoptotic pathways in A549 and H460 cells by blocking the cell cycle and down-regulating cyclin D1 and cyclin dependent kinases (CDKs) expression^48^,^49^. In A549 cells, simvastatin-induced cell death was associated with decreased expression of survivin^50^, like in breast cancer cells.

Our previous investigations in human airway smooth muscle and fibroblasts and in human atrial fibroblasts showed that simvastatin induces the intrinsic apoptosis pathways in a small GTPase prenylation dependent way. This statin effect was not reversible with cholesterol co-treatment^27^,^28^,^51^. It has been also shown that GGTI-298, but not FTI-277, can mimic the cytotoxic effects of statins, indicating that statins induce cell death by inactivating Rho/Rac GTPase activities^52^. These results were also confirmed by translocation of RhoA, Rac1/2/3, and Cdc42 to cytosol in simvastatin-treated cells. In eukaryotic cells, prenylation is carried out by three different prenyl transferases: farnesyl transferase (FT), geranylgeranyl transferase I (GGTI) and Rab geranylgeranyl transferase (Rab GGT or GGTII)^53^. FT is responsible for prenylation of proteins such as Ras and lamins. The GGTI catalyses the geranylgeranylation of proteins in the Rho and Rac family, whereas the Rab GGT is responsible for the geranylgeranylation of the Rab protein family^54^. Simvastatin increases Rac GTP loading in THP-1 monocyte cells while decreasing prenylation of Rac in the presence of amyloid beta stimulation, and decreasing the induction of inflammation in these cells^55^. In addition, T-cell function is not affected by Rho GTP loading, whereas geranylgeranylation of these small Rho GTPases is the determining step that affects their function^56^,^57^. Our results showed that statin-induced Rho protein GTP loading is dependent to specific cell type and Rho protein localization and geranylgeranylation is the determining step in regulation of their function (Figs 5, 6 and 7).

One of the signaling proteins involved in transmitting extracellular stimuli to intracellular components is the Ras (Rat sarcoma) superfamily of small GTPases^5^,^58^. In normal cellular and biological conditions these proteins play essential functions in the regulation of pathways critical to cytoskeletal reorganization, cell survival and proliferation, transformation, and vesicular trafficking^59^,^60^. One of the major subgroups in Ras superfamily of small GTPases is Rho (Ras homologous) family GTPases^61^. The members of small Rho GTPase (Rho, Rac, and Cdc42) are well known for their key functions in regulating actin cytoskeleton controlling actin stress fibers^62^,^63^,^64^. The function of small GTPases is very tightly regulated by different molecular switches, including prenylation and guanosine triphosphate (GTP) binding. When bound to GTP and prenylated, small GTPases not only translocate to the membrane but also undergo a conformational change to engage effectors that promote downstream signaling pathways^5^,^65^.

In addition to their role in normal physiological and developmental processes, Rho GTPases can contribute to pathological processes including cancer cell migration, invasion, metastasis, and inflammation^66^,^67^. Activating mutations in Ras proteins (such as K-Ras, N-Ras, and H-Ras) are found in 15 to 30% of human tumors^68^. Several Rho proteins are upregulated in different human tumor types, including RhoA^69^,^70^, RhoC^71^, Rac1^69^,^70^, Rac2^72^, Rac3^73^, and Cdc42^69^,^70^. Rac1 is overexpressed in breast^70^, lung^74^, oral squamous cell^75^, testicular^76^, and gastric carcinoma^77^, whereas RhoA is also overexpressed in breast^70^, colon^78^, lung^64^, gastric^77^, head and neck^79^, bladder^80^, and testicular carcinomas^76^,^77^,^79^,^80^,^81^. Cdc42 is overexpressed in breast^69^ and testicular cancers^76^. Therefore, inhibition of these over-expressed Rho small GTPases might improve current therapeutic approaches in the treatment these cancers.

There is increasing interest in repurposing statins for use in the treatment of human cancers. An epidemiologic study has shown that statin use in patients with cancer was associated with reduced cancer-related mortality^82^. Our results showed that the HMGCR inhibitor simvastatin induces intrinsic apoptotic cell death in different cancer cell models via a unique small Rho GTPase-dependent pathway which prevents small Rho GTPase prenylation and inhibits subsequent translocation to the membrane, thus, effectively deactivating Rho GTPases. Interestingly, our current investigation suggests that cholesterol depletion is not involved in simvastatin-induced apoptosis in glioblastoma, neuroblastoma, non-small lung cancer cells, and breast cancer cell lines.

In our forthcoming studies, we are studying how the combination of simvastatin with different chemotherapeutic agents may synergize to enhance cancer cell killing. We will explore mechanisms of cell fate that could mediate this beneficial statin effect, thereby supplementing and enhancing the therapeutic effects of cancer medications.

## Materials and Methods

### Reagents

Cell culture plastic ware, media, penicillin, streptomycin, and fetal bovine serum (FBS) were obtained from VWR (Toronto, ON, Canada). Secondary anti-rabbit and anti-mouse antibodies, propidium iodide (PI), simvastatin, mevalonate (Mev), farnesylpyrophosphate (FPP), geranylgeranylpyrophosphate (GGPP), GGTi-298, FTi-277, cholesterol, and 3-(4,5-dimethyl-2-thiazolyl)-2,5-diphenyl-2H-tetrazolium bromide) (MTT), were obtained from Sigma (Sigma-Aldrich, Oakville, CA). Rabbit anti-human/mouse Rac1/2/3, RhoA, and Cdc42 were purchased from Cell Signaling (Canada). Casapase-Glo^®^-3/7, Caspase-Glo^®^-8 and Caspase-Glo^®^-9 assay were purchased from Promega (Toronto, ON, Canada). Tetramethylrhodamine, Methyl Ester, Perchlorate (TMRM) was purchased from Biotium (Hayward, CA, USA). Thin layer chromatographic plates (silica gel G, 0.25-mm thickness) were from Fisher (Winnipeg, Manitoba, Canada). Ecolite scintillant was from ICN Biochemicals (Montreal, Quebec, Canada). Unlabeled cholesterol standard was from Sigma-Aldrich (Oakville, ON, Canada). [1-^14^C]Acetate was from American Radiolabeled Chemicals (St. Louis, MO, USA). All other biochemicals were American Chemical Society grade and were obtained from either Sigma-Aldrich or Fisher Scientific (Winnipeg, MB, Canada).

### Cell Culture

For all experiments we used the following human cancer cell lines: (U87 (ATCC- HTB-14™), U251 (ATCC)), neuroblastoma (SH-SY5Y (ATCC-CRL-2266™)), lung adenocarcinoma (A549 (ATCC- CCL-185™), H460 (ATCC- HTB-177™), H1650 (ATCC-CRL-5883™), H1975 (ATCC-CRL-5908™)), breast cancer (MCF-7 (ATCC-HTB-22™), MDA-MB-231 (ATCC-HTB-26™)), human astrocyte (Sciencell-1800), human HBE1(Gift from Dr. Amir Zeki lab, UC Davis), and human MCFD10A (Gift from Dr. Amir Zeki lab, UC Davis). Cancer cells were cultured in DMEM (high glucose) supplemented with FBS (10%), penicillin (1%), and streptomycin (1%). Human astrocytes were cultured in DMEM supplemented with FBS (10%), penicillin (1%), and streptomycin (1%). HBE1 cells were grown to 90% confluency on a 100 mm cell culture plate in serum-free medium containing Ham’s F12/DMEM (1:1), 15 mM NaHCO_3_, 15 mM Hepes (pH 7.4), with these factors: transferrin (5 μg/mL), insulin (5 μg/mL), cholera toxin (10 ng/mL), epidermal growth factor (10 ng/mL), dexamethasone (0.1 μM), bovine hypothalamus extract (15 μg/mL). Cells were then transferred to 6-well plates in submerged media conditions. MCF10A were grown in Lonza CC-3150 MEGM per ATCC’s recommendation without gentamicin/amphotericinplus 100 ng/mL cholera toxin. Cells were then transferred to 6-well plates.

### Cell Viability Assay

We measured the viability of different cancer cells under various treatment conditions, as described previously, using MTT assay^28^,^83^,^84^,^85^. Briefly, U87, U251, SH-SY5Y, A549, H460, H1650, H1975, MCF7, MDA-MB-231n normal astrocytes, HBE1, and MCF10A cells were treated with different concentrations of simvastatin for different time points (0–20 μM, 0–96 h). Relative cell viability (percent of control) was calculated using the equation: (mean OD _(570)_ of treated cells/mean OD _(570)_ of control cells) × 100. For each time point, the treated cells were compared with control cells that had been treated with dimethyl sulfoxide (DMSO) vehicle only. In experiments investigating the role of MEV, GGPP, FPP, and cholesterol in mediating the cytotoxic effects of simvastatin on tumor cells, MEV (2.5 and 5 mM), GGPP (7.5 and 15 μM), FPP (7.5 and 15 μM), and cholesterol (25 and 50 μM) were added to culture media 4 h before subsequent simvastatin co-treatment (10 μM, 96 h). The cytotoxic effect of GGTi-298 and FTi-277 (0–40 μM) in U87, A549, and MDA-MB-231, was evaluated using the same protocol as described for the simvastatin-induced cytotoxic assay.

### Analysis of cellular morphology

To assess cell viability based on gross cellular appearance, U87, A549, and MDA-MB-231 cells were grown on 6 well plates, treated with simvastatin (10 μM, 60 h) and assessed by phase contrast microscopy (Zeiss Axioverts 100) using a Olympus DP10 CCD digital camera to capture images^27^.

### Measurement of Apoptosis by Flow Cytometry

The Nicoletti method was used to measure cellular apoptosis^86^,^87^. Briefly, cells cultured in 12 well plates were treated with simvastatin (10 μM, 48 h). Cells were detached using EDTA buffer and harvested by centrifugation at 1500 g for 5 min at 4 °C. Cells were washed once in PBS before resuspending in a hypotonic PI lysis buffer (0.1% Triton X-100, 1% sodium citrate, 0.5 mg/ml RNase A, 40 μg/ml propidium iodide). Cell nuclei were incubated at 37 °C for 30 min and analyzed by flow cytometry. Apoptotic nuclei were located on the left side of the G1 peak and contained hypo-diploid DNA.

### Luminometric Caspase Assay

For the proteolytic activity of caspase-8 (IETD-ase), -3/-7 (DEVD-ase), -9 (LEHDase), Caspase-Glo^®^-3/-7, -8, and -9 (Promega) were determined in luminometric assays according to the manufacturer’s instructions and our previous report^51^,^87^. Briefly, cells were grown in 96-well plate (15,000 cells/well) and treated with simvastatin (10 μM, 36 h). Fresh caspase reagents were prepared containing z-LETD-Luciferin, z-DEVD-Luciferin or z-LEHD-Luciferin and whole protein cell lysate extract buffer. Cells treated only with medium and reagent blank (negative controls) were included in each experiment. Plates were shaken gently at 300–500 rpm for 30 sec and then incubated for 90 min at room temperature. The solution was transferred to a white-well plate and then the luminescence was measured for each sample and compared to negative control values^51^,^87^.

### TMRM Staining for Mitochondrial Membrane Potential Measurement

U87, A549, and MDA-MB231 cells were cultured in 6 well plates and were treated with simvastatin (10 μM, 36 h) and then were stained with (Tetramethylrhodamine, Methyl Ester, Perchlorate TMRM (100 nM) and Hoechst (10 μM) nuclear stain at 37 °C for 30 minutes. After washing, the cells they were imaged using florescence microscope and the fluorescence was quantified on ImageJ software in at least 50 individual cells in different views (NIH, Bethesda, MD, USA)^88^.

### Membrane anchoring of Rho GTPases

To determine membrane anchoring of prenylated Rho and Rac GTPases, U251, A549, and MDA-MB-231 were cultured in DMEM with high glucose/10% FBS in the presence or absence of simvastatin (10 μM, 36 h). Upon washing, cells were scraped in ice cold buffer (10 mM Tris–HCl, pH 7.5, 0.1 mM EDTA, 0.1 mM EGTA, 1 mM dithiothreitol, and protease inhibitor cocktail), sonicated on ice three times for 5 sec and cell homogenate was separated into cytoplasmic and membrane fractions by ultracentrifugation (100,000 × g for 35 min)^89^. Membrane fractions were solubilized in dissociation buffer (50 mM Tris–HCl, pH 7.5, 0.15 M NaCl, 1 mM dithiothreitol, 1% SDS, 1 mM EDTA, 1 mM EGTA, protease inhibitor cocktail), and subsequently size fractioned by SDS–PAGE (15%) for immunoblot analysis using primary antibodies to Rac1/2/3, Cdc42, and RhoA (all Cell Signaling)^27^.

### Measurement of Rho GTPase activity

Rho-GTP bound was measured in snap-frozen cell lysates harvested from cells cultured in medium without FBS and treated with simvastatin (10 μM), mevalonate (2.5 mM), FPP, and GGPP (15 μM), Simva. + mevalonate, simvastatin + FPP, and simvastatin + GGPP for 36 h. We used a luminometric-based G-LISA Rho –GTP bound assay (Cytoskeleton, Inc, Denver, Colo) for U251, A549, and MDA-MB231 cells Briefly, cell lysates were subjected to Rho binding domain in a Rho-GTP affinity 96-well plate (Cytoskeleton, Inc, Denver, Colo). Rho-GTP was detected with specific primary antibody, followed by horseradish peroxidase-conjugated secondary antibody detection and development with a chemiluminescent reagent. A constitutively active Rho-GTP provided in the kit was used as positive control in all experiments^90^.

### Cholesterol mass measurement

U87, A549, and MDA-MB231 cells were treated with simvastatin (10 μM, 36 h) and subsequently cholesterol content of cells was determined by colorimetric reaction using the Amplex Red Cholesterol assay kit (Invitrogen) as per the manufacturer’s instructions. Lipids were extracted and cholesterol isolated on thin layer chromatography plates prior to analysis as described^27^,^91^,^92^,^93^. All isolates were measured immediately after drying down under N_2_.

### Cholesterol labeling experiment

Cells were incubated with 0.1 μM [1-^14^C]acetate (10 μCi/dish) for 24 h. Lipids were extracted and cholesterol isolated on thin layer chromatography plates as above. Spots corresponding to cholesterol standards were removed, and radioactivity incorporated into cholesterol determined by liquid scintillation counting as described^92^.

### Immunoblotting

Western blotting was used to detect Cdc42, Rac1/2/3 and RhoA in membrane and cytosolic fractions as described previously^51^,^94^. Pan-cadherin and GAPDH were used to confirm membrane and cytosolic fraction purity, respectively. Briefly, cells protein extracts were prepared in lysis buffer (20 mM Tris-HCl (pH 7.5), 0.5 mM PMSF, 0.5% Nonidet P-40, 100 μM β-glycerol 3-phosphate and 0.5% protease inhibitor cocktail). Supernatant protein content was measured by Lowry protein assay after centrifugation at 13,000 g for 10 min. Proteins were separated by SDS-PAGE and transferred to nylon membranes under reducing conditions. Membranes were blocked with non-fat dried milk and Tween 20 followed by overnight incubation with the primary antibodies at 4 °C, followed by incubation with HRP-conjugated secondary antibody for 1 h at room temperature. Blots were developed by enhanced chemiluminescence (ECL) detection (Amersham-Pharmacia Biotech).

### Statistical Analysis

The results were expressed as means ± SEM and statistical differences were evaluated by one-way or two-way ANOVA followed by Tukey’s or Bonferroni’s post hoc testing, using Graph Pad Prism 7.0. A p-value < 0.05 was considered statistically significant. For all experiments data were collected in five replicates and in three separate experiments.

## Additional Information

**How to cite this article**: Alizadeh, J. *et al*. Mevalonate Cascade Inhibition by Simvastatin Induces the Intrinsic Apoptosis Pathway via Depletion of Isoprenoids in Tumor Cells. *Sci. Rep.* **7**, 44841; doi: 10.1038/srep44841 (2017).

**Publisher's note:** Springer Nature remains neutral with regard to jurisdictional claims in published maps and institutional affiliations.

## Supplementary Material

Supplementary Information
