# Supplementary material for: Mevalonate Cascade Inhibition by Simvastatin Induces the Intrinsic Apoptosis Pathway via Depletion of Isoprenoids in Tumor Cells
Source: Sci Rep. 2017 Mar 27;7:44841. doi: 10.1038/srep44841 (PMC5366866; doi:10.1038/srep44841)
Supplement: Supplementary Information [file srep44841-s1.doc]

**Mevalonate Cascade Inhibition by Simvastatin Induces the Intrinsic Apoptosis Pathway via Depletion of Isoprenoids in Tumor Cells**

Javad Alizadeh^1,2^, Amir A. Zeki^3^, Nima Mirzaei^1^, Sandipan Tewary^1^, Adel Rezaei Moghaddam^1,2^, Alexandra Glogowska^1^, Pandian Nagakannan^4^, Eftekhar Eftekharpour^4^, Emilia Wiechec^5^, Joseph W. Gordon^1^, Fred. Y. Xu^6^, Jared T. Field^7^, Ken Y. Yoneda^3^, Nicholas J Kenyon^3^, Mohammad Hashemi^8^, Grant M. Hatch^6^, Sabine Hombach-Klonisch^1^, Thomas Klonisch^1^, Saeid Ghavami^1,2,9^

^1^Department of Human Anatomy and Cell Science, Faculty of Health Sciences, College of Medicine, University of Manitoba, Winnipeg, Canada

^2^Biology of Breathing Theme, Children Hospital Research Institute of Manitoba, University of Manitoba, Canada

^3^Division of Pulmonary, Critical Care, and Sleep Medicine, Department of Internal Medicine, Center for Comparative Respiratory Biology and

Medicine, Davis, CA, USA

^4^Regenerative Medicine Program and Spinal Cord research Center, Faculty of Health Science, University of Manitoba

^5^Department of Clinical and Experimental Moledicine, Division of Otorhinolaryngology, Linköping University, 581-85 Linköping, Sweden

^6^DREAM, Children’s Hospital Research Institute of Manitoba, Center for Research and Treatment of Atherosclerosis and Department of Pharmacology and Therapeutics, University of Manitoba, Winnipeg, Manitoba

^7^Department of Biological Sciences, Faculty of Science, University of Manitoba

^8^Department of Clinical Biochemistry, Zehedan University of Medical Sciences, Zahedan, Iran

^9^Health Policy Research Centre, Shiraz University of Medical Sciences, Shiraz Iran

**Address for Correspondence:**

Saeid Ghavami, PhD

Department of Human Anatomy and Cell Science, Faculty of Health Sciences, College of Medicine, University of Manitoba, Winnipeg, Canada

Tel: Office, +1 204, 272 3061, Lab, +1 204 272 3071

Email: [saeid.ghavami@umanitoba.ca](mailto:saeid.ghavami@umanitoba.ca)

**Key Words**: Simvastatin, non-small cell lung cancer, breast cancer, glioblastoma, neuroblastoma, Rho GTPase, cancer cell biology

**Supplementary Figure 1**: **Simvastatin induces cell death in glioblastoma, neuroblastoma, non-small lung cancer cell, breast cancer cell lines and their corresponding non-malignanat cells. (A, B)** U251 cells were treated with simvastatin (1, 2.5, 5, 10 or 20 μM) and cell viability was assessed 48 and 96 h thereafter by MTT assay. Control cells for each time point were treated with the solvent control (DMSO). Results are expressed as percentage of corresponding time point control and represent the means ± SD of 15 replicates in three independent experiments (**, *P*<0.01; ***, *P*<0.001). **(C, D)** SH-SY5Y cells were treated with simvastatin (1, 2.5, 5, 10 or 20 μM) and cell viability was assessed 48 and 96 h thereafter by MTT assay. Control cells for each time point were treated with the solvent control (DMSO). Results are expressed as percentage of corresponding time point control and represent the means ± SD of 15 replicates in three independent experiments (***, *P*<0.001). **(E, F)** Human astrocytes cells were treated with simvastatin (1, 2.5, 5, 10 or 20 μM) and cell viability was assessed 48 and 96 hrs thereafter by MTT assay. Control cells for each time point were treated with the solvent control (DMSO). Results are expressed as percentage of corresponding time point control and represent the means ± SD of 15 replicates in three independent experiments (**, *P*<0.01; ***, *P*<0.001). **(G, H)** H460 cells were treated with simvastatin (1, 2.5, 5, 10 or 20 μM) and cell viability was assessed 48 and 96 h thereafter by MTT assay. Control cells for each time point were treated with the solvent control (DMSO). Results are expressed as percentage of corresponding time point control and represent the means ± SD of 15 replicates in three independent experiments (*, *P*<0.1; ***, *P*<0.001). **(I, J)** H1650 cells were treated with simvastatin (1, 2.5, 5, 10 or 20 μM) and cell viability was assessed 48 and 96 h thereafter by MTT assay. Control cells for each time point were treated with the solvent control (DMSO). Results are expressed as percentage of corresponding time point control and represent the means ± SD of 15 replicates in three independent experiments (**, *P*<0.01; ***, *P*<0.001). **(K, L)** H1975 cells were treated with simvastatin (1, 2.5, 5, 10 or 20 μM) and cell viability was assessed 48 and 96 h thereafter by MTT assay. Control cells for each time point were treated with the solvent control (DMSO). Results are expressed as percentage of corresponding time point control and represent the means ± SD of 15 replicates in three independent experiments (**, *P*<0.01; ***, *P*<0.001). **(M, N)** HBE1 cells were treated with simvastatin (1, 2.5, 5, 10 or 20 μM) and cell viability was assessed 48 and 96 h thereafter by MTT assay. Control cells for each time point were treated with the solvent control (DMSO). Results are expressed as percentage of corresponding time point control and represent the means ± SD of 15 replicates in three independent experiments (**, *P*<0.01; ***, *P*<0.001). **(O, P)** MCF7 cells were treated with simvastatin (1, 2.5, 5, 10 or 20 μM) and cell viability was assessed 48 and 96 h thereafter by MTT assay. Control cells for each time point were treated with the solvent control (DMSO). Results are expressed as percentage of corresponding time point control and represent the means ± SD of 15 replicates in three independent experiments (**, *P*<0.01; ***, *P*<0.001). **(Q, S)** MCF10A cells were treated with simvastatin (1, 2.5, 5, 10 or 20 μM) and cell viability was assessed 48 and 96 h thereafter by MTT assay. Control cells for each time point were treated with the solvent control (DMSO). Results are expressed as percentage of corresponding time point control and represent the means ± SD of 15 replicates in three independent experiments (**, *P*<0.01; ***, *P*<0.001).

**Supplementary Figure 2: Simvastatin induces intrinsic apoptosis in glioblastoma, neuroblastoma, non-small lung cancer cell, and breast cancer cell lines.** Percent sub-G1 (**A**) U251, (**B**) SH-SY5Y, (**C**) H460, (**D**) H1650, (**E**) H1975, (**F**) MCF-7 abundance induced by simvastatin (10 μM) or DMSO solvent control after 60 hrs. Results represent the means ± SD of 9 replicates in three independent experiments. *, *P*<0.05; and ***, *P*<0.001 compared to time-matched control. Effects of simvastatin (10 μM) treatment (36 hrs) on caspase-8, caspase-3/-7, and caspase-9 enzymatic activity, as detected by Caspase-Glo® luminometric assay in (**G**) U251, (**H**) SH-SY5Y, (**I**) H460, (**J**) H1650, (**K**) H1975, (**L**) MCF-7. Caspase activity normalized to that measured for solvent-only treated cultures is represented on the Y-axis. The data represent mean ± SD of triplicate experiments performed on 3 independent experiments.

**Supplementary Figure 3: Simvastatin-inducds cell death in glioblastoma, neuroblastoma, non-small lung cancer cell, and breast cancer cell lines is independent of cholesterol.**

2.5, and 5 mM MEV or , 25, 50 µM cholesterol, were added to the cells 4 hrs prior to treatment with simvastatin (10 μM, 96 hrs) and cell death was measured by MTT assay in, U251 (**A, B**), SH-SY5Y (**C, D**), H460 (**E, F**), H1650 (**G, H**), H1975 (**I, J**), and MCF-7 (**K, L**). For each experiment control cells were treated with simvastatin solvent (DMSO) alone (control) or with both DMSO and the appropriate solvent (i.e. ethanol for “mevalonate control”). Results are expressed as mean ± SD of 9 replicate in 3 independent experiments (*, P<0.05, **, P<0.01, and ***, *P*<0.001).

**Supplementary Figure 4: Simvastatininduced cell death in glioblastoma, neuroblastoma, non-small lung cancer cell, and breast cancer cell lines is dependent on FPP and GGPP.**

7.5, 15 μM FPP, or 7.5, 15 μM GGPP were added to the cells 4 hrs prior to treatment with simvastatin (10 μM, 96 hrs) on cell death, measured by MTT assay in, U251 (**A, B**), SH-SY5Y (**C, D**), H460 (**E, F**), H1650 (**G, H**), H1975 (**I, J**), and MCF-7 (**K, L**). For each experiment control cells were treated with simvastatin solvent (DMSO) alone (control) or with both DMSO and the appropriate solvent for each cholesterol precursor (i.e. DMSO for “FPP control” and “GGPP control”). Results are expressed as mean ± SD of 9 replicate in 3 independent experiments (*, P<0.05, **, P<0.01, and ***, *P*<0.001).
